# *FMR1 *CGG allele size and prevalence ascertained through newborn screening in the United States

**DOI:** 10.1186/gm401

**Published:** 2012-12-21

**Authors:** Flora Tassone, Ka Pou Iong, Tzu-Han Tong, Joyce Lo, Louise W Gane, Elizabeth Berry-Kravis, Danh Nguyen, Lisa Y Mu, Jennifer Laffin, Don B Bailey, Randi J Hagerman

**Affiliations:** 1 Department of Biochemistry and Molecular Medicine, UC Davis, Sacramento, CA 95817, USA; 2 MIND Institute, UC Davis Medical Center, Sacramento, CA 95817, USA; 3 Department of Pediatrics, Neurological Sciences, and Biochemistry, Rush University Medical Center, Chicago, IL 60612, USA; 4 Division of Biostatistics, UC Davis, Davis, CA 95616, USA; 5 Department of Pediatrics, University of Wisconsin, Madison, WI 53706, USA; 6 RTI International, Research Triangle Park, NC 27709, USA; 7 Department of Pediatrics, UC Davis, Sacramento, CA 95817, USA

## Abstract

**Background:**

Population screening for *FMR1 *mutations has been a topic of considerable discussion since the *FMR1 *gene was identified in 1991. Advances in understanding the molecular basis of fragile X syndrome (FXS) and in genetic testing methods have led to new, less expensive methodology to use for large screening endeavors. A core criterion for newborn screening is an accurate understanding of the public health burden of a disease, considering both disease severity and prevalence rate. This article addresses this need by reporting prevalence rates observed in a pilot newborn screening study for FXS in the US.

**Methods:**

Blood spot screening of 14,207 newborns (7,312 males and 6,895 females) was conducted in three birthing hospitals across the United States beginning in November 2008, using a PCR-based approach.

**Results:**

The prevalence of gray zone alleles was 1:66 females and 1:112 males, while the prevalence of a premutation was 1:209 females and 1:430 males. Differences in prevalence rates were observed among the various ethnic groups; specifically higher frequency for gray zone alleles in males was observed in the White group compared to the Hispanic and African-American groups. One full mutation male was identified (>200 CGG repeats).

**Conclusions:**

The presented pilot study shows that newborn screening in fragile X is technically feasible and provides overall prevalence of the premutation and gray zone alleles in the USA, suggesting that the prevalence of the premutation, particularly in males, is higher than has been previously reported.

## Background

Fragile X syndrome (FXS), the most common single gene cause of inherited intellectual disabilities and autism, is characterized by a CGG-repeat expansion (>200 CGG repeats, full mutation) in the portion of the first exon of the fragile X mental retardation 1 gene (*FMR1*), which encodes the 5' UTR of the *FMR1 *mRNA. When the full mutation is present, epigenetic modification of the CGG rich region turns off the gene, which results in absence or deficit of the encoded product, FMRP, leading to defects in synaptic plasticity. *FMR1 *premutation carriers have an unstable expansion containing 55 to 200 CGG repeats and gray zone or intermediate allele carriers have small expansions of 45 to 54 repeats [1].

The *FMR1 *full mutation can cause a broad spectrum of involvement, including intellectual disability, behavior problems, social deficits and autism spectrum disorders (ASD) [2-4]. Significant clinical involvement has also been reported in some premutation carriers, including medical, neurological and psychiatric problems such as ASD, attention deficit-hyperactivity disorder (ADHD), depression and anxiety [5-12]. Moreover, fragile X-associated primary ovarian insufficiency (FXPOI) occurs in approximately 20% of female carriers [13,14] and fragile X-associated tremor ataxia syndrome (FXTAS) affects approximately 40% of older male carriers, and approximately 8 to 16% of older female carriers [8,15-17]. Risks associated with gray zone or intermediate alleles still need to be verified, but these alleles may be associated with an increased risk for FXTAS and FXPOI, and can be unstable when transmitted across generations [18-21].

The reported prevalence of the full mutation in the general population ranges from 1:2,500 to 1:8,000 in females and approximately 1:4,000 to 1:5,000 in males [22-28]. Premutation carriers (55 to 200 CGG repeats) are more common, with estimates ranging between 1:130 and 1:256 for females and 1:250 and 1:813 for males [27,29-34]. Several studies suggest that FXS prevalence rates may differ across ethnic groups and countries based on studies of populations in the United Kingdom [25], Spain [30], Finland [35], Asia (Taiwan [36,37], Japan [38]), Israel [26,39-41], and North America [29,42,43]. However, discerning the 'true' incidence rate has been challenging, due primarily to small sample sizes and some design limitations, such as selection bias in studies that focus on specialized populations (for example, children in special education settings [44], pregnant volunteer adults with no history of mental retardation [41] or adults with no major health problems [38]). Further complicating this picture is the varying definition of CGG size ranges for intermediate/gray alleles and premutation alleles. A summary of the studies estimating prevalence since 1995 in various populations, designs, and settings is shown in Table 1, while the prevalence of *FMR1 *expanded alleles from newborn screening studies conducted in different countries is summarized in Table 2.

**Table 1:** Prevalence data in general population.

Reference	Location	Number tested	Gender	Genotype	CGG range	Prevalence
[42]	Canada	10,624	Female	Pre	55-101	1/259
[81]	USA	3,345	Pregnant/non-pregnant women	Gray	40-49	1/52 (no fhx)
						1/107 (fhx)
					50-59	0/474 (no fhx)
						0/214 (fhx)
				Pre	60-200	1/158 (no fhx)
						0/214 (fhx)
				Full	>200	0/474 (no fhx)
						0/214 (fhx)
[25]	UK, 11-16 years	347		Fragile X		1/2,720
[35]	Finland	1,738	Pregnant women	Pre	60-200	1/246
				Full	>200	0/1,477
[40]	Israel	10,587	Female	Gray/Pre	51-200	1/77
[82]	Israel	9,660	Pregnant/non-pregnant women	Pre	50-199	1/114
				Full	>200	0/9,660
[26]	Israel	9,459	Pregnant/non-pregnant women	Pre	52-199	1/73
				Full	>200	1/2,365
[83]	UK	3,738	Male	FRAXA full	≥200	1/187
[84]	Finland	239	Pregnant women	Pre	61-200	1/220
				Full	>200	0/220
[41]	Israel	14,334	Pregnant/non-pregnant women	Pre	55-200	1/113
				Full	>200	1/4,778
[29]	Canada	10,572	Male	Pre	55 to <230	1/813
				Full	>230	1/155
[24]	USA	2,250	Male	Full	≥200	1/353
				Intermediate	41-60	1/27
		1,089	Female	Pre	61-199	1/531
				Intermediate	41-60	1/19
[85]	Taiwan	1,002	Pregnant women	Gray	40-52	1/46
				Pre	>52	0/1,002
[33]	USA	29,103	Pregnant women	Gray	45-54	1/143
				Pre	55-200	1/382
				Full	>200	0/2,292
[39]	Israel	40,079	Pregnant/non-pregnant women	Pre	55-199	1/158 (no fhx)
						1/150 (fhx*)
				Full	>200	1/36,483 (no fhx)
						1/899 (fhx)
[86]	Australia	338	Non-pregnant women	Gray	45-54	1/22
				Pre	55-200	1/65
				Full	>200	0/65
[34]	Canada	21,411	Female	Gray	45-54	1/86
				Pre	55-200	1/241
[38]	Japanese	576	Female	Intermediate	40-50	1/324
		370	Male	Intermediate	40-50	1/103
[32]	USA	11,759	Female from cystic fibrosis screening	Pre	55-200	1/245
		2,011	Ashkenazi Jewish women	Pre	55-200	1/134
[74]	USA	3,273	Male	Gray	45-54	1/42
				Pre	55-200	1/468
		3,474	Female	Gray	45-54	1/35
				Pre	55-200	1/151

fhx, family history of FXS.

* Family history of individuals with intellectual disability, developmental problems, or autism in extended family but without relatives who were fragile X carriers.

**Table 2:** Prevalence data from newborn screening studies

Reference	Location	Ethnicity	Number tested	Gender	Genotype	CGG range	Number positive	Prevalence
[23]	Georgia, USA	45% Caucasian	36,124	Male	Full	>200	7	-
		30% African-American						
		15% Hispanic						
		2% Asian						
		2% Multicultural						
		1% American Indian						
		5% Unknown						
[37]	Taiwan	Asian	4,843	Male	Gray	40-54	90	-
					Pre	55-200	2	-
					Full	>200	2^a^	-
[87]	Canada	Canadian	1,000	Male	Gray	40-60	51^b^	-
			1,000	Female	Pre	>60	1^c^	-
[30]	Spain	Hispanic	5,267	Male	Gray	45-54	199	1/26
					Pre	55-200	21	1/251
					Full	>200	2	1/2633
[51]	Catalan, Spain	Hispanic	5,000	Male	Gray	53-55	11	1/449
					Pre	56-200	4	1/1233
					Full	>200	2	1/2466
[43]	South Carolina, USA	NA^d^	1,459	Male	Pre	55-200	2	1/730
					Full	>200	2	1/730
[36]	Taiwan	Asian	10,046	Male	Gray	45-54	70	1/143
					Pre	55-200	6	1/1674
					Full	>200	1^e^	-

^a^Need Southern blot confirmation. ^b^Number positive includes both genders. ^c^Gender of the positive premutation is male. ^d^Racial data not collected for this study. ^e^Not confirmed.

A large-scale population-based screening for FXS, in both males and females across the entire spectrum of fragile X mutations, has not been conducted in the United States. One problem has been the lack of a molecular test capable of identifying *FMR1 *alleles throughout the range (from normal to the full mutation) in both males and females. In recent years, several methodologies have been published and claimed to be suitable for large population screening [22,30,45-50], although all have presented some technical and non-technical problems, including the amount of DNA template required, degradation due to the use of bisulfite, inclusion of females, and failure to detect unmethylated expanded alleles. Importantly, no study in both genders, across all the mutation ranges, has been conducted on blood spot cards, a central requirement for newborn screening. The few large studies that have been conducted on blood spot cards include a study of 36,154 de-identified blood spot cards from male newborns, targeting only those with a methylated full mutation [22] and reports on newborns from Spain and from Taiwan that also included only males (Table 2) [30,36,37,51].

Traditionally, Southern blot analysis has been considered the most accurate method to size the full mutation and to determine the methylation status of the expanded alleles for all mutation sizes. However, it is laborious, expensive and requires a large amount of DNA, making it poorly suitable for screening purposes. Screening of blood spot cards by a PCR-based method is the best approach currently available for screening large populations. However, because PCR testing can report CGG repeat lengths for all size ranges, clinicians and policy makers associated with newborn screening will need to consider which categories of *FMR1 *expansions to report. In part this decision will be determined by the clinical utility of the information and associated ethical issues. However, more accurate estimates of prevalence are essential so that the public health burden (for example, counseling and treatment costs, patient education before screening) can be assessed more accurately.

To help answer this question, we report here the outcomes of a large fragile X newborn screening study conducted in the United States, consisting of 14,207 newborn blood spot samples (7,312 males and 6,895 females). The screening method utilized allowed for precise quantification of CGG allele size, distribution of allele sizes within different ethnic groups and determination of the prevalence of gray zone and premutation alleles in both males and females. The advantages of the screening approach used in the present study, in addition to its high throughput ability, are the ability to detect expanded alleles throughout the range in both genders, the use of blood spot cards for the screening, and the relatively unbiased population sample that should yield representative allele frequencies for different ethnic groups in the USA. The sample size is too small to provide an estimate of full mutation prevalence, and thus the paper is focused on gray zone and premutation alleles. These alleles are much more common than full mutation alleles and their disclosure complicates the counseling burden that would result. We also report the prevalence for an expanded gray zone allele range, from 40 to 54 CGG repeats for comparison with other studies that have reported allele frequencies using this expanded size range [52,53].

## Materials and methods

### Study subjects

Bloodspots from newborns at UC Davis Medical Center (UCDMC, Sacramento, CA, USA), Rush University Medical Center (RUMC, Chicago, IL, USA) and the University of North Carolina (UNC) Hospital (Chapel Hill, NC, USA) were made from extra blood at the time of the state-mandated heel stick. Babies did not receive an extra heel stick if there was not enough blood from the mandated state newborn screen heel stick already available to obtain the extra card. At all three sites a research assistant reviewed the newborn nursery admittance record daily, approached parents to obtain consent for the newborn to participate in the fragile X screening program, which was separate from the state newborn screening programs. They entered the patient's room and asked for permission to speak with the family. If the parents decided not to speak to the research assistant, their refusal was noted. When permission was given by the parents for the research assistant to speak with them, a prepared script, institutional review board (IRB) approved, was used to briefly introduce the purpose of the study. The parents were asked if they had any questions and if they would like to participate in the formal consenting process. The reason(s) as to why a family did or did not choose to participate were recorded when possible.

#### University of North Carolina Hospital

At the UNC site, consent was obtained prior to the heel stick for the state screening and collection of the extra blood spot card for fragile X screening. Only blood spot cards from consented newborns were included in the study. Cards were shipped in the initial period of this project, to the UCD MIND Institute Molecular Laboratory in Sacramento and later to the Wisconsin State Health Department Cytogenetics and Molecular Laboratory for CGG allele size analysis. Only families of infants in the regular care nursery were approached. The screening involved an informed consent under a protocol approved by the UNC IRB. A description of the screening process, participation rates, and reasons for accepting or declining screening has been previously reported [54].

#### Rush University Medical Center (Chicago, IL)

At RUMC it was not possible to obtain the state screening after consent due to the phlebotomy schedule. Consequently, the extra spot was obtained when the state screening heel stick was done and consenting was done afterwards to request use of the blood spot for the research project. This avoided the need to do a second heel stick on the babies. Consent forms used were approved by the RUMC IRB. For consenting families, demographic information was obtained from the family after the consent was signed. The bloodspot was identified by the newborn's last name, gender and date of birth. All data were recorded in computer files at RUMC, and then the blood spots were shipped to the UCD MIND Institute Molecular Laboratory in Sacramento for the CGG allele size analysis. The blood spots collected from families who chose not to participate in the newborn screening study but did not object to anonymous screening, were de-identified and sent to the UCD MIND Institute Molecular Laboratory. Specifically, non-consenting parents were told verbally that the blood spot would be used for anonymous population screening to obtain information on allele prevalence; if the parent objected, the sample was discarded. Families of infants from both regular care and special care nurseries were approached to participate in the study.

#### UC Davis Medical Center (Sacramento, CA)

A similar procedure was followed at the UCDMC site. An additional spot was obtained when the state screening heel stick was done and consenting was carried out with a UC Davis IRB approved consent form. Only families of infants in the regular care nursery were approached. Blood spot cards from consented newborns were included; however a previous anonymous screening was allowed by the UC Davis IRB using a different funding source and before funding for consented screening was obtained; thus, the anonymous screening was also included at the UCDMC site for the prevalence figures described below. For those who did not sign consent, but allowed anonymous screening, or for those who were not approached, bloodspots were assayed as anonymous screening. These latter bloodspots were stripped of all identifiers and patient codes, preserving only stated gender and ethnicity of the donor, to ensure that the samples were not traceable to the newborn. Those who specifically denied consent were not included in this study. To each bloodspot card a local accession number was assigned and underwent genotyping analysis.

### Follow up for infants carrying an expanded allele

At each site the family was contacted by phone following the identification of a consented newborn with the premutation or full mutation. The results were conveyed and explained to the parents, questions answered, and a visit was scheduled for the child to be seen for further medical follow-up and a genetic counseling session. The expanded allele was confirmed by standard *FMR1 *diagnostic testing (including Southern blot analysis) on a confirmatory blood sample from the infant, in a Clinical Laboratory Improvement Accreditation (CLIA) College of American Pathologists (CAP) certified clinical diagnostic laboratory at UCDMC, RUMC, or UNC. In all cases, expanded premutation alleles identified through newborn screening were confirmed by standard *FMR1 *diagnostic testing.

### Bloodspot screening: CGG sizing

Most of the samples were collected on FTA cards (Whatman Inc., Piscataway, NJ, USA); however, blood spots collected between January and May 2012 were collected on 903 paper (Whatman Inc.) at RUMC and at UCDMC. Blood spot cards were used directly in the PCR mixtures after being washed with FTA purification reagents (Qiagen, Valencia, CA, USA) as previously described [50] or DNA was isolated from two to three punches using either a QIAxtractor (Qiagen) or a Biomek NX workstation (Beckman Coulter Inc., Brea, CA, USA) as described below. No differences were obtained in terms of DNA quality or yield from either FTA or 903 cards.

### DNA isolation from bloodspot punches

Isolation of DNA was performed using the Agencourt Genfind v2 DNA Isolation Kit (Beckman Coulter Inc.) on the Biomek NX workstation (Beckman Coulter Inc.) following the manufacturer's instructions. Briefly, each blood spot sample was lysed with 150 µl of lysis buffer with 3 µl of proteinase K followed by incubation with 75 µl of binding buffer. Samples were then washed twice and eluted with 30 µl of nuclease-free water. The isolation procedure followed Agencourt Genfind v2 FTA Cards software (Beckman Coulter Inc.) with a minor change of replacing Wash 2 solution with 70% ethanol. Isolated DNA was stored at -20°C. Isolation of DNA was also performed using the QIAxtractor Reagent Pack (Qiagen) on the QIAxtractor (Qiagen) following the manufacturer's instructions. Each blood spot sample was lysed with 280 µl lysis buffer with 20 µl of proteinase K followed by incubation with 600 µl of binding buffer. Samples were then washed twice with wash solution (DXW) and final wash solution (DXF) and eluted with 60 µl of nuclease-free water. The isolation procedure followed the QIAxtractor software (Qiagen). The isolated bloodspot DNA was stored at -20°C.

### PCR analysis

The bloodspot PCR screening approach was as follows: first round PCR screening was used to size all normal, intermediate and/or premutation alleles using c and f primers (by Fast Start approach, CGG rich or Expand Long PCR; Roche Diagnostics, Indianapolis, IN, USA). Male samples with no band on the first round or female samples with a single band underwent a second PCR screening assay using a CCG chimeric primer [50,55].

Genomic DNA was amplified using Fast Start PCR protocol (Roche Diagnostics). Master mix containing primers c and f was prepared and used according to the manufacturer's instructions; primers c and f yield amplicons of 221+ (CGG)n bp. PCR reactions were run in the Applied Biosystems 9700 thermocycler with PCR conditions as previously described [30]. The PCR products were analyzed using the ABI 3730 Capillary Electrophoresis (CE) Genetic Analyzer (Applied Biosystems, Foster City, CA, USA). Unpurified PCR product (2 μl) was mixed with 12 μl of Hi-Di Formamide (Applied Biosystems) and 2 μl of a ROX 1000 Size Ladder (Asuragen Inc., Austin, TX, USA). Samples were heat-denatured at 95°C for 2 minutes followed by cooling on ice before being transferred to the CE instrument. Samples that did not yield a band for males and yielded only one band for females after the first PCR round were subjected to a secondary CGG-primer-based PCR screening [50,55]. Samples were prepared for the PCR with a master mix from AmplideX *FMR1 *reagent kit (Asuragen Inc.) containing *FMR1 *For, Rev FAM primers and *FMR1 *CGG primer or by using the CGG rich approach (Roche Diagnostics). PCR conditions were as indicated by the manufacturer (Asuragen Inc.) and were as previously described [50,55]. The PCR products were run on CE for detection as previously described [45]. Serial peaks were visualized on CE with the CGG-chimeric primer when an expanded allele was present. CE data were analyzed by the ABI Genescan analysis software.

### Statistical analysis

Student's *t*-test and ANOVA were used to compare CGG distribution for gender and ethnicity. Exact confidence intervals were obtained for overall prevalence estimates, as well as among ethnicity groups across gender. Comparisons of prevalence were based on Fisher's exact test. Association between ethnicity and consenting status was analyzed using logistical regression. SAS version 9.2 (Cary, NC) was utilized for the analysis.

## Results

### Study population

A total of 14,207 blood spot samples, 7,312 males and 6,895 females, from newborns were collected across the three sites from November 2008 through May 2012. The study population included five ethnic groups (based on mother's ethnicity): White/Caucasian (White; N = 4,161, 29.4%), Hispanic/Latino (Hispanic; N = 3,493, 24.6%), African American/Black (Black; N = 3,069, 21.6%), Asian/Indian (Asian; N = 796, 5.6%), and Others, including Native American (Others; N = 1,286, 9.1%). There were 1,374 subjects (9.7%) from whom ethnicity could be not ascertained.

### CGG allele size distribution

The CGG screening was conducted following the workflow previously described in Tassone *et al*. [50]. Briefly, male and female newborns that generated, respectively, a single or two bands (two alleles) after the first PCR *FMR1 *specific screening (using primers c and f) were not analyzed further. Blood spots were run twice if they failed to amplify the first time. All samples included in the analysis generated clear amplified *FMR1 *specific products. Females with only one amplified band and males without a clear amplified PCR band (one case of a full mutation male newborn identified in this study) underwent the second screening PCR using a CGG primer as previously described [50,55]. Of the remaining 20,930 alleles, 20,710 had a CGG repeat number within the normal range (CGG range 6 to 44); 170 (105 females and 65 males) were gray zone alleles (mean CGG = 48 in both genders, CGG range 45 to 54); 50 (33 females and 17 males) harbored a premutation allele (mean CGG = 70 in both females and males, CGG range was 55 to 130). Additionally, 21 males generated 2 bands after the first PCR screening and 6 females were not definitely genotyped and therefore were excluded from the analysis. Although some of those samples may have been mislabeled with respect to the sex of the newborn, some could have been subjects with Klinefelter Syndrome, but they were not studied further because of study and IRB constraints. Among the 14,207 newborns screened, one male (7,312 total males screened) was identified as having a full mutation allele at UCDMC. This subject was not included in the subsequent prevalence analysis.

**Figure 1:**
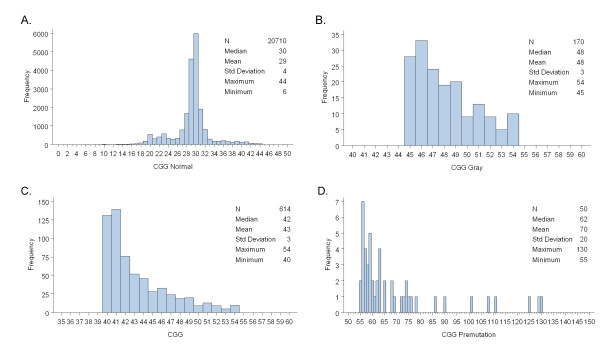
**CGG repeat allele size distribution**. Histograms display the CGG repeat length observed in the newborn screening by allele category. A) *FMR1 *alleles in the normal range (<45 CGG repeats, n = 20710 alleles). B) *FMR1 *alleles in the gray zone range (45-54 CGG repeats, n = 170 alleles). C) *FMR1 *alleles in the expanded gray zone range (40-54 CGG repeats, n = 614 alleles). D) *FMR1 *alleles in the premutation range (55-200 CGG repeats, n = 50).

There was no gender difference in CGG distribution for either gray (female: N = 105, mean 48, standard deviation (SD) 3; male: N = 65, mean 48, SD 3; *P *= 0.3829) or premutation alleles (female: N = 33, mean 70, SD 21; male: N = 17, mean 70, SD 17; *P *= 0.9453). Results are shown in Table 3. CGG allele size distribution is represented in Figure 1a for N = 20,710 alleles (7,208 from male, 13,502 from both female alleles); the observed CGG range is from 6 to 44, with a median of 29 (SD ± 4) and mode of 30. For the 170 gray zone alleles in the 45 to 54 range (65 males and 105 females; median 48; SD ± 3) CGG size distribution is shown in Figure 1b. Because some studies have reported the 40 to 54 CGG range as an expanded gray zone range [52,53], we also examined the CGG allele distribution in the 614 alleles in this range (383 were females, 4 of which had both alleles with a CGG repeat number between 40 and 54; 227 were males; median 42; SD ± 3; Figure 1c). For premutation carriers (CGG 55 to 200), Figure 1d displays CGG repeats for 50 individuals with observed CGG repeat length ranging from 55 to 130 (17 males and 33 females; median 62; SD ± 20) with the majority of the subjects (n = 35, 70%) carrying an allele with repeat number <70 CGG.

**Table 3:** Summary of CGG distribution across gender in the three categories (normal, gray zone, premutation)

Gender	N	Mean	SD	Median
**Normal**				
F	13,502^a^	29	4	30
M	7,208	29	4	29
				
**Gray**				
F	105	48	3	47
M	65	48	3	48
				
**Pre**				
F	33	70	21	60
M	17	70	17	68

^a^Both alleles from normal female subjects are included. F, female; M, male.

We determined the CGG size distribution of gray zone and premutation alleles across different ethnic groups. Results show that, among premutation carriers, Whites tended to have slightly higher CGG repeat size (mean 76, SD ± 24, N = 16) than other ethnicity groups (mean ranging from 62 to 75), although the difference did not reach statistical significance. It should be noted that these observed differences should be considered descriptive due to the small sample size (Table 4).

**Table 4:** Summary of CGG distribution across ethnicity groups in the three categories

	N	Mean	SD
**Combine**			
Normal^a^	20,710	29	4
Gray	170	48	3
Pre	50	70	20
			
**White/Caucasian**			
Normal^a^	6,044	29	4
Gray	70	48	3
Pre	16	76	24
			
**African-American/Black**			
Normal^a^	4,506	29	4
Gray	31	48	2
Pre	11	71	21
			
**Hispanic/Latino**			
Normal^a^	5,126	29	4
Gray	38	48	3
Pre	6	75	26
			
**Asian, including Indian**			
Normal^a^	1,147	30	3
Gray	5	47	1
Pre	4	62	9
			
**Other, including Native American**			
Normal^a^	1,864	29	4
Gray	14	48	3
Pre	9	63	6
			
**Unknown**			
Normal^a^	2,023	29	4
Gray	12	50	3
Pre	4	57	1

^a^Both alleles from normal female subjects are included.

### Prevalence

Across the three sites, the prevalence for gray zone alleles was 1:66 in females (95% confidence interval (CI) 1:80 to 1:54); and 1:112 (95% CI 1:145 to 1:88) in males. In the 40 to 54 expanded gray zone CGG range, the prevalence was 1:18 (95% CI 1:20 to 1:16) in females and 1:32 (95% CI 1:37 to 1:28) in males. The prevalence for premutation alleles was 1:209 (95% CI 1:303 to 1:149) in females and 1:430 (95% CI 1:736 to 1:268) in males, which translates into a male to female prevalence rate of 1 to 2.05. The prevalence for both gray zone and premutation alleles for each site is reported in Table 5.

**Table 5:** Summary of prevalence across and over all sites

			Gray		Pre	
				
Site	Gender	Total	N	Prevalence	N	Prevalence
CH	F	3,140	45	1 : 70	12	1 : 262
	M	3,279	25	1 : 131	4	1 : 820
NC	F	1,754	29	1 : 60	11	1 : 159
	M	1,861	23	1 : 81	4	1 : 465
SAC	F	1,995	31	1 : 64	10	1 : 200
	M	2,150	17	1 : 126	9	1 : 239
Overall	F	6,889	105	1:66	33	1:209
	M	7,290	65	1:112	17	1:430

CH, Chicago, RUMC; F, female; M, male; NC, North Carolina, UNC; SAC, Sacramento, UCDMC.

We also obtained estimates of the prevalence of gray zone alleles in different ethnic groups. Although the sample size was small, we also report the observed premutation allele prevalence within ethnicity groups. The observed premutation prevalence in females who were Black (1:168) was higher compared to females who were Hispanic (1:570, *P *= 0.0785) but this was not a significant difference. The observed premutation prevalence in males who were Black (1:780) was lower compared to those who were White (1:358) and those who were Hispanic (1:595). The observed prevalence of gray zone alleles in White males (1:61) was significantly higher than in black males (1:142, *P *= 0.0153), and Hispanic/Latino males (1: 198, *P *= 0.0007). The observed prevalence of gray zone alleles was similar across White (1:58), Black (1:75) and Hispanic groups (1:59) in females. We did not compare the prevalence among other ethnic groups because the sample size was too small (Tables 6 and 7).

**Table 6:** Prevalence of grayzone and premutation alleles in females and males across ethnic groups

		Overall	White	Black	Hispanic	Asian	Other	Unknown
		Group* N = 14,179	N = 4,161	N = 3069	N = 3493	N = 796	N = 1286	N = 1374
**Females (N)**		6,889	2,014	1,508	1,709	368	614	676
**Normal (count)**		6,751	1969	1,479	1,677	360	601	665
Gray	Count (rate)	105 (1:66)	35 (1:58)	20 (1:75)	29 (1:59)	5 (1:74)	8 (1:77)	8 (1:84)
	(95% CI**) Gray	1:80-1:54	1:82-1:42	1:123-1:49	1:88-1:41	1:226-1:32	1:177-1:39	1:195-1:43
Premutation	Count (rate)	33 (1:209)	10 (1:201)	9 (1:168)	3 (1:570)	3 (1:123)	5 (1:123)	3 (1:225)
	(95% CI) Pre	1:303-1:149	1:420-1:110	1:366-1:89	1:2761-1:195	1:594-1:42	1:377-1:53	1:1092-1:77
**Males (N)**		7,290	2,147	1,561	1,784	428	672	698
**Normal (count)**		7,208	2,106	1,548	1,772	427	662	693
Gray	Count (rate)	65 (1:112)	35 (1:61)	11 (1:142)	9 (1:198)	0 (N/A)	6 (1:112)	4 (1:174)
	(95% CI) Gray	1:145-1:88	1:88-1:44	1:284-1:80	1:433-1:105	N/A	1:305-1:52	1:640-1:68
Premutation	Count (rate)	17 (1:429)	6 (1:358)	2 (1:780)	3 (1:595)	1 (1:428)	4 (1:168)	1 (1:698)
	(95% CI) Pre	1:736-1:268	1:974-1:165	1:6443-1:216	1:2882-1:204	1:16906-1:77	1:616-1:66	1:27570-1:126

Group* refers to the total number of males and females for all ethnicities. CI** refers to Confidence Interval.

**Table 7:** *P*-value based on Fisher exact test (2 by 2 table)

	Female		Male	
		
	Gray	Pre	Gray	Pre
White versus Black	0.3409	0.817	0.0153	0.4805
White versus Hispanic	1	0.161	0.0007	0.5239
Black versus Hispanic	0.4712	0.0785	0.5051	1

## Discussion

In the United States, newborn screening is an important state-based public health program that began over 40 years ago with the development of a screening test for phenylketonuria using newborn bloodspots dried onto a filter paper card [56,57]. Many factors could influence a decision to include a given condition in a newborn screening program, such as the severity of the condition, the availability of effective treatment, the age of onset, and the complexity, availability or cost of the test [58]. Fragile X screening has captured increasing attention lately for both potential benefits and concerns that affect the development of a screening program. Fragile X screening was not recommended for newborn screening in the American College of Medical Genetics report of 2006 [59] primarily because of the lack of an accurate screening test and the absence of data on benefits at that time. In the past few years the advent of clinical trials of targeted treatments for FXS and indications of positive outcomes in early phase studies [60-64] have been exciting developments that promote the need for newborn screening for FXS. Some of the targeted treatments and additional interventions are being studied in children in the toddler period and these interventions will likely enhance the developmental/behavioral interventions for young children [65]. In addition, the development of a new PCR-based screening approach utilized here has further stimulated the discussion around newborn screening in fragile X.

Accurate estimates of frequency of *FMR1 *mutations in the general population are needed to better estimate fragile X allele frequencies for all racial and ethnic groups and to determine the ramifications of any population screening program in terms of numbers of identified cases. The increasing number of disorders attributed to the premutation has also encouraged better epidemiology data. Indeed, great interest has been focused on premutation carrier detection, since premutation alleles have been found to be associated with FXPOI [13,14,66] and FXTAS [67-69] and sometimes with neurodevelopmental disorders, such as ASDs and ADHD [5,9,70], which can respond to treatments [71].

Here, we report allele frequency distributions found in a pilot newborn screening study from three sites in the US, using a novel PCR-based approach to demonstrate the feasibility of screening for *FMR1 *mutations in a large sample size and with samples collected on blood spot cards. This is the largest newborn sample size screened in the US for both males and females and for the detection of expanded alleles throughout the normal to full mutation range. We found that the most common alleles were those containing 29 and 30 CGG repeats, regardless of ethnicity, in agreement with previous reports. The screening identified 170 newborns carrying a gray zone allele (45 to 54 CGG repeats) with a prevalence of 1:66 in females and 1:112 in males. Some studies [52,53] have advocated for expanding the gray zone to 40 to 54 CGG repeats because there is an elevation in the *FMR1 *mRNA expression levels in this range and there may be evidence of risk of clinical involvement, including an increased rate of primary ovarian insufficiency (POI) compared to the general population [18,19]. In addition, an increased prevalence of gray zone alleles has also been recently reported in subjects with parkinsonism [52,72] and several cases of FXTAS have been reported in gray zone [20,73]. Thus, we also report the prevalence in this expanded gray zone range as 1:32 in males and 1:18 in females based on the total number of newborns screened. Our findings regarding the prevalence of the premutation alleles (1:209 in females and 1:430 in males) are within the range of what was previously reported in females [29], but in males we observed a prevalence almost two-fold higher than that in the Canadian study (1:813) [29], lower than in the Spanish population [30] but in line with a recent population-based screening study of older adults in Wisconsin, US (1:468 in males) [74]. It is interesting to note that from our study the female to male prevalence rate for the premutation is 2.05, in agreement with the predicted ratio described by Hagerman [31]. Although the size of the premutation alleles varied between 55 and 130 CGG repeats in females and between 56 and 125 CGG repeats in males, it is interesting to note that 70% of the premutation alleles contained <70 CGG repeats, in agreement with a recent report [32]. This may be of relevance for estimating the frequency of *FMR1 *related disorders in the general population since individuals with >70 repeats are more likely to have premutation disorders [75]. If we consider that the prevalence of a premutation allele in males is approximately 1:400 and if FXTAS is affecting approximately 40% of the premutation male carriers, then we would expect that 1.6 males out of 2,000 in the general population would develop the neurodegenerative syndrome. As was described in a recent study [76], FXTAS is far less likely in patients with <70 repeats. Thus, despite rare reports of FXTAS in the gray zone [52] and in the low end of the premutation range, it is likely the frequency of FXTAS in the general population is lower than 1.6/2,000. However, mild neurological problems, such as neuropathy or balance problems associated with the premutation, are likely to be close to this prevalence and more common than in those with a definitive diagnosis of FXTAS.

Only one male newborn, out of the total 7,312 males screened, was found to have a full mutation at the UCDMC site. A large screening of newborns (n = 36,154) reported a prevalence of 1:5,161 in males [23]; however, our sample size is too small to be confident of a prevalence estimate for the full mutation. Indeed, one would need in excess of 70,000 samples to estimate a prevalence of 1:5,000 and 95% CI within a 50% margin of error.

Although the CGG size distribution did not show a difference between the two genders and among different ethnic groups, differences were detected in the prevalence of expanded alleles. Specifically, the prevalence of gray zone alleles was higher in White males compared to Black and Hispanic males. Differences in the prevalence between the different ethnic groups were also observed for the premutation alleles; however, they did not reach statistical significance likely due to the small number. It is important to consider the potential difference in prevalence of premutation alleles in different populations as this could explain both the differences in premutation prevalence and the incidence of FXS among different studies.

Identifying and reporting babies with a premutation is somewhat controversial, with important arguments on both sides of the equation. One argument in favor of disclosure is the potential benefit for extended family members, in terms of genetic and reproductive counseling. Some of these family members may be suffering from clinical problems related to the premutation or full mutation segregating in the family, and can benefit from knowledge of their condition to help direct treatment [77]. Identification of babies with the premutation can also lead to early intervention or treatment when needed with appropriate follow-up [71]. Although premutation babies are far less likely to show developmental problems than full mutation babies, some are at risk for learning problems, ASD, or seizures, and early intervention will be important to implement if developmental problems emerge in follow-up [5,9,70,71].

On the negative side of identifying *FMR1 *premutation carriers at the time of birth is that the family is told of possible future problems related to the premutation that may or may not develop, including FXTAS, and this may cause excessive worries for the family, especially since the certainty of problems will be unknown. Many families may not want to know about carrier status, and a robust consent process is needed to assure that families understand the kind of information that could be learned from FX screening. The high rate of carrier detection makes clear the burden that screening would place on genetic counseling.

The identification of a newborn with the premutation or the full mutation can create the need for cascade testing throughout the family. Some family members will be interested in knowing if they are carriers, especially if they have medical problems that may relate to premutation involvement. These types of problems include depression, anxiety [12,78,79], autoimmune problems, such as fibromyalgia or hypothyroidism [8,11], hypertension [80], sleep apnea [10], neuropathy, FXPOI and FXTAS. In our study, the largest family so far identified through cascade testing after the newborn was identified as a carrier had 16 additional carriers identified, including a great grandmother with probable FXTAS [77], several great aunts with neurological problems, others with emotional difficulties and female carriers with significant needs for reproductive counseling. Although it is unclear whether all of these problems are a direct result of the premutation alone, it is clear that there is a need to test extended family members in relation to premutation and full mutation disorders. However, the time and energy of the counseling and health care professionals for cascade testing of identified families may be a limiting factor on how many individuals in one family tree can be identified.

## Conclusions

This study demonstrates that newborn screening is technically feasible, and advances our understanding of the overall prevalence of the premutation and gray zone alleles in the USA and their prevalence in different ethnic groups. It also suggests that the prevalence of the premutation in both males and females is higher than was found in a previous large study in North America [29]. In addition, this study provides the expected approximately 2:1 ratio of female to male carriers [31]. Clearly, newborn screening using a methodology that detects CGG repeats will result in the identification of many more premutation than full mutation babies. Before newborn screening for fragile X mutations is expanded nationally, further work is needed to understand the impact that identification of the premutation has on families; the developmental trajectories of children with the premutation; the possible need for a robust consent process; and ultimately whether the nation's public health system has the capacity to address the counseling and educational needs that inevitably will arise.

## Abbreviations

ADHD: attention deficit-hyperactivity disorder; ASD: autism spectrum disorder; CE: Capillary Electrophoresis; CI: confidence interval; FXPOI: fragile X-associated primary ovarian insufficiency; FXS: fragile X syndrome; FXTAS: fragile X-associated tremor ataxia syndrome; IRB: institutional review board; RUMC: Rush University Medical Center; SD: standard deviation; UCDMC: UC Davis Medical Center; UNC: University of North Carolina; UTR: untranslated region.

## Competing interests

RJH has received funding to carry out treatment trials in fragile X syndrome or autism from Roche, Novartis, Seaside Therapeutics, Forest, and Curemark. She has also consulted with Roche and Novartis regarding targeted treatments in fragile X syndrome. The remaining authors have no competing interests to declare.

## Authors' contributions

TF designed the study, drafted the manuscript and participated in the data analysis and interpretation of the results. IKP, TT, LJ and LJ carried out the molecular screening. GLW helped with the recruitment and data analysis. B-KE drafted the manuscript and participated to the data analysis and interpretation of the results. DVN and MY performed the statistical analysis and interpretation of the result, contributed to drafting the manuscript, and managed data quality and analysis. BDB drafted the manuscript and participated in the data analysis. RJH drafted the manuscript and participated in the data analysis and interpretation of the results. All authors read and approved the final manuscript.
